# Quantifying mechanical loading and elastic strain energy of the human Achilles tendon during walking and running

**DOI:** 10.1038/s41598-021-84847-w

**Published:** 2021-03-12

**Authors:** Mohamadreza Kharazi, Sebastian Bohm, Christos Theodorakis, Falk Mersmann, Adamantios Arampatzis

**Affiliations:** 1 Department of Training and Movement Sciences, Humboldt-Universität zu Berlin, Philippstr. 13, Haus 11, 10115 Berlin, Germany; 2 Berlin School of Movement Science, Berlin, Germany

**Keywords:** Physiology, Bone quality and biomechanics

## Abstract

The purpose of the current study was to assess in vivo Achilles tendon (AT) mechanical loading and strain energy during locomotion. We measured AT length considering its curve-path shape. Eleven participants walked at 1.4 m/s and ran at 2.5 m/s and 3.5 m/s on a treadmill. The AT length was defined as the distance between its origin at the gastrocnemius medialis myotendinous junction (MTJ) and the calcaneal insertion. The MTJ was tracked using ultrasonography and projected to the reconstructed skin surface to account for its misalignment. Skin-to-bone displacements were assessed during a passive rotation (5°/s) of the ankle joint. Force and strain energy of the AT during locomotion were calculated by fitting a quadratic function to the experimentally measured tendon force–length curve obtained from maximum voluntary isometric contractions. The maximum AT strain and force were affected by speed (p < 0.05, ranging from 4.0 to 4.9% strain and 1.989 to 2.556 kN), yet insufficient in magnitude to be considered as an effective stimulus for tendon adaptation. Besides the important tendon energy recoil during the propulsion phase (7.8 to 11.3 J), we found a recoil of elastic strain energy at the beginning of the stance phase of running (70–77 ms after touch down) between 1.7 ± 0.6 and 1.9 ± 1.1 J, which might be functionally relevant for running efficiency.

## Introduction

Tendons cannot generate force actively, yet their elastic behavior upon loading influences the muscle–tendon unit's function during locomotion. The human Achilles tendon (AT) length changes during functional tasks as walking, running, jumping, or cycling is important to understand the interaction between muscle and tendon^1–3^, assess tendon loading, and examine tendon's elastic strain energy^4–6^. The AT's reported maximum strain values during running are between 4.6 and 9.0%^7–9^ and between 4.0 to 4.3% during walking^4,7^. Corresponding AT force values ranged from 3.06 to 4.64 kN during running^10,11^ and about 2.63 kN during walking^12^. These results suggest substantial mechanical loading of the AT during human locomotion. The AT can adapt to external mechanical loading by increasing its stiffness, elastic modulus and size^13,14^. Loading-induced alteration of tendon properties is a biological mechanism to maintain the functional integrity of the muscle–tendon unit and to keep tendon mechanical loading in a physiological range during functional tasks^15,16^. Repetitive loading of the AT with a strain magnitude between 4.5 and 6.5% has been evidenced as an effective mechanical stimulus, improving AT mechanical properties^17–19^. This tendon strain range is commonly reached at about 90% of a voluntary maximum isometric contraction of the adjacent muscle, which results in AT forces between 2.34 and 3.69 kN^18–20^.

Although the above mentioned reports of AT strain and force during running indicate sufficient AT loading for the initiation of adaptive alterations in tendon properties^7–10^, most studies, which compared the AT mechanical properties between runners and untrained controls were not able to detect any differences between the two groups^20–23^. Furthermore, in the longitudinal study of Hansen et al., no significant changes in AT's mechanical properties were observed after nine months (78 sessions) of running training^24^. In addition to a shorter loading duration (i.e., during running) that may explain this discrepancy (estimates of loading vs. lack of adaptive response)^17–19^, these findings might also result from the methodological approaches used in vivo assessment of AT strain during locomotion. Previous in vivo approaches used for assessing AT length during functional tasks either calculated AT length using a simple planimetric model^7,8^ or did not consider the AT's concave curvature in their measurements^1,25,26^. There is evidence that considering the AT as a straight line between calcaneus (insertion) and gastrocnemius medialis (GM) myotendinous junction (MTJ, origin) results in an underestimation of the AT length and substantial errors (up to 78%) of the AT length changes^27^.

**Figure 1:**
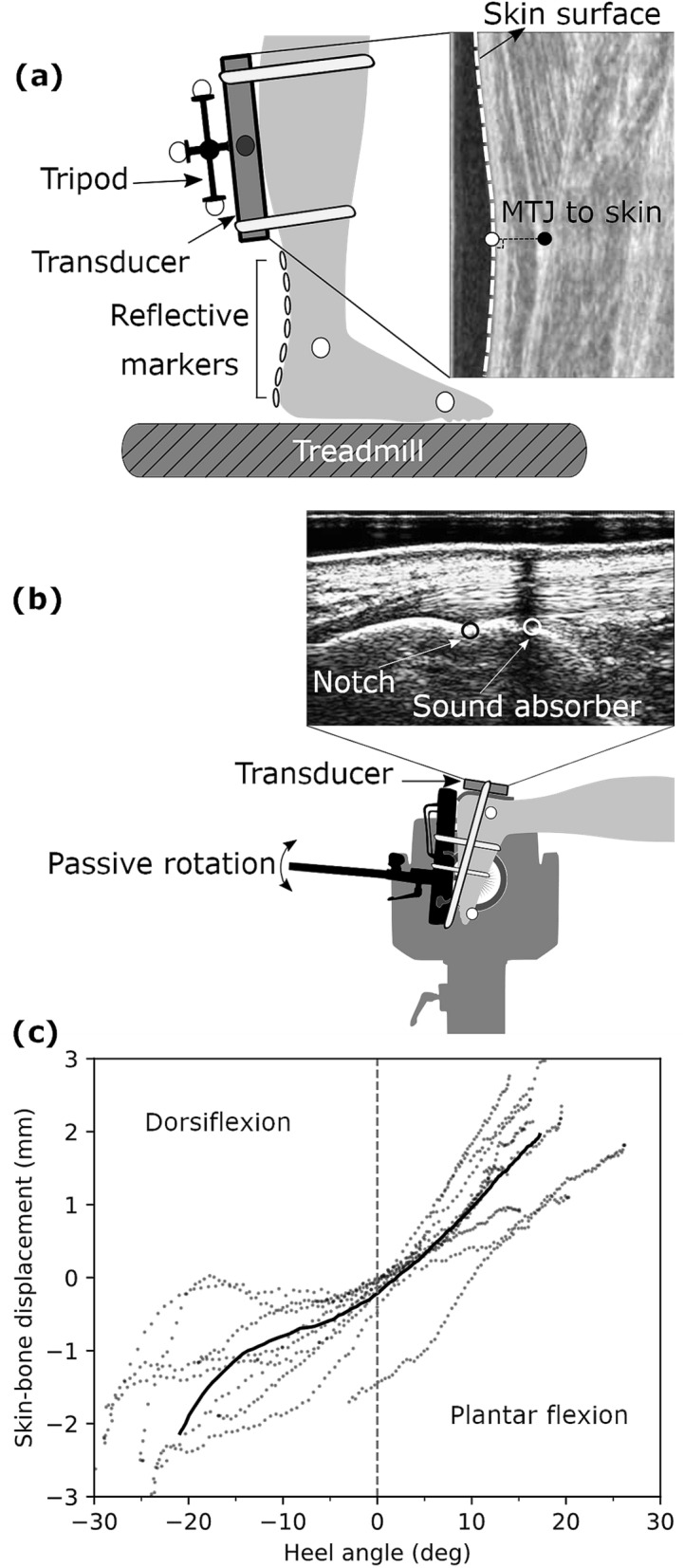
(**a**) Experimental setup for determining Achilles tendon (AT) length during gait. (**a**) Reflective foil markers on the skin were used to reconstruct the curve-path shape of the AT. The position of the gastrocnemius medialis myotendinous junction was projected to the skin surface, and the coordinates of the ultrasound images were transformed to the global coordinate system using a marker tripod attached to the ultrasound probe. (**b**) A three-centimeter ultrasound probe was placed on top of the calcaneus bone and a sound-absorptive marker in-between to measure the differences in calcaneus bone displacements (notch) and the skin (absorber marker) displacements as a function of the heel to shank angle. (**c**) Average (solid line) and individual (filled dots) data of skin-to-bone displacements vs. heel angle. Positive heel angles represent plantar flexion, and negative ones are dorsiflexion (eleven participants with three repetitions).

The instantaneous curved length of the AT can be obtained using a line of reflective markers attached to the skin from the tuber calcanei to the MTJ of the GM^28,29^. Although this method has been validated by comparing the outcomes with accurate AT length measurements from magnetic resonance imaging^30^, it has not been applied to assess AT length during locomotion. The neglect of AT's curvature during locomotion could result in a 3.4 mm error when the ankle rotates from 30° in plantar flexion to 15° in dorsiflexion^27^. Considering an average AT rest length of 200 mm would cause a significant strain error of 1.7%. During dynamic functional tasks, two more issues may introduce errors in such AT length measurements. First, the original position of the GM MTJ is not aligned with the reflective markers attached to the skin surface and, therefore, for an accurate AT length measurement, the identified position of the MTJ should be projected to the skin (Fig. 1a). Secondly, the attached marker at the calcaneus that defines the AT insertion represents the underlying bones' movement. However, the relative movement of the skin to the bone can introduce important artifacts in the measurements and should be considered. Therefore, the skin's potential displacement relative to the bone underneath the calcaneus marker that defines the AT insertion can also introduce errors in the AT length measurement^1^.

The elastic strain energy recoil of the AT during the propulsion phase of walking and running is a well-known mechanism within the muscle–tendon unit, which increases the efficiency of muscle output power^4–6^. The contribution of the elastic strain energy recoil to the muscle–tendon unit's positive work is greater compared to the work produced by the muscle fascicles alone^4–6^. However, there are also indications of AT elastic strain energy recoil during the early stance phase of running^31^, which is not well understood. Komi et al.^31^, using an implanted transducer around the AT, measured in vivo AT forces during running and found an apparent decrease of AT force after heel contact, particularly in rearfoot runners, indicating an energy recoil of the AT directly after touchdown. Considering that in this phase, the fascicles of the triceps surae muscles actively shorten^7,9,32^, it can be argued that the contractile elements do not absorb this elastic strain energy recoil from the AT and, thus, it might be an additional important source of energy for human running.

The current study aimed to assess the AT's mechanical loading and strain energy during walking and running. For this purpose, we measured the AT length during walking and running using a new in vivo approach, which considers the tendon curve-path shape using skin markers, taking into account the projection of the MTJ to the skin surface as well as potential movements of the skin relative to the calcaneus bone. Tendon force and strain energy were then calculated based on an experimentally determined tendon force–elongation relationship. We hypothesized a relevant contribution of the MTJ projection to the skin and skin-to-bone displacement on the AT length. Further, we expected lower levels of tendon strain during locomotion, as previously reported, insufficient in magnitude to serve as a stimulus for the adaptation of AT mechanical properties. Finally, we hypothesized a functional relevant recoil of tendon elastic strain energy to the body at the beginning of the stance phase during running.

## Results

**Figure 2:**
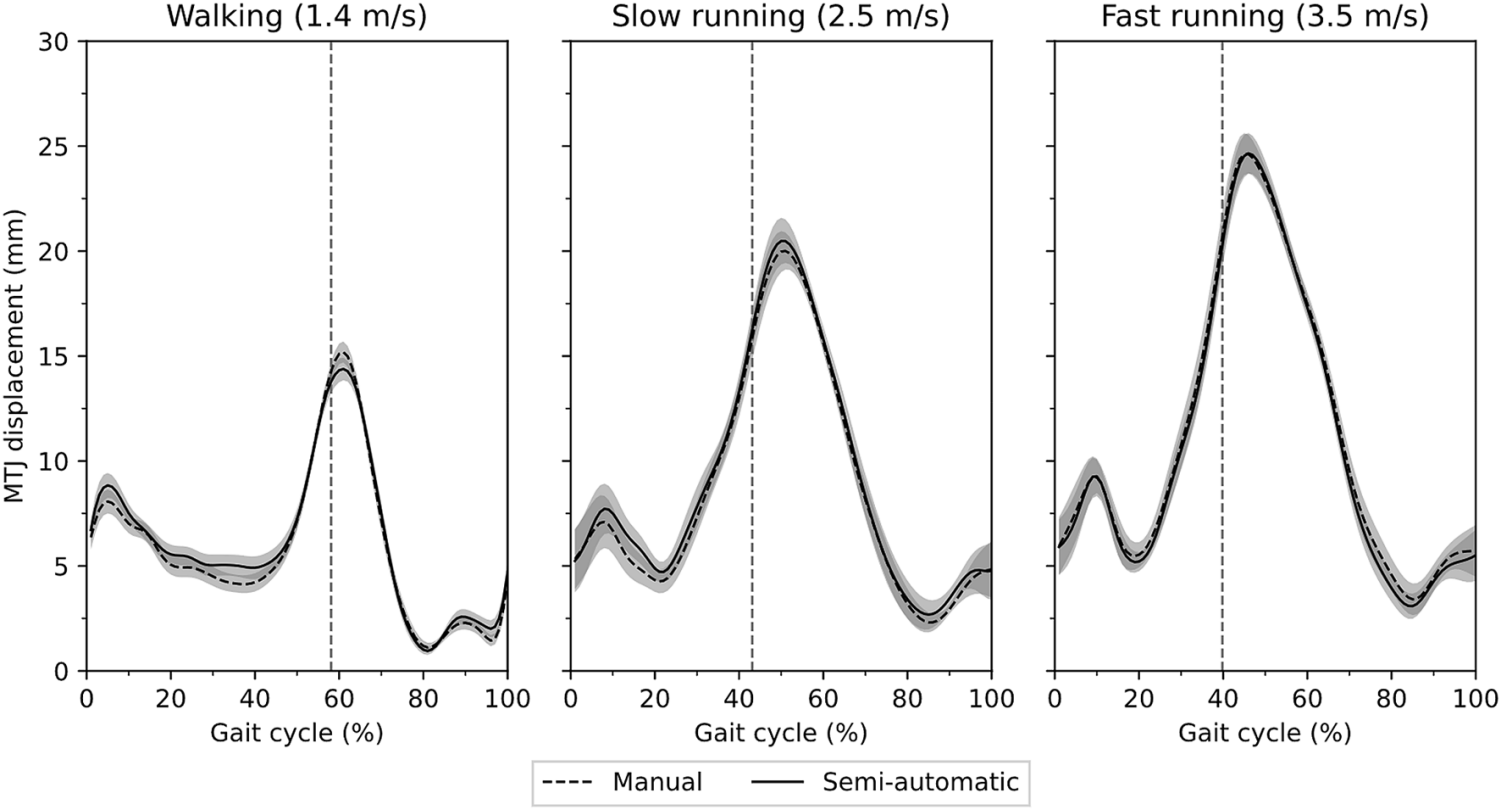
Displacement of the gastrocnemius medialis myotendinous junction using the self-developed semi-automatic algorithm compared to the manual tracking for walking, slow running, and fast running. The x-axis is normalized to the gait cycle. The gray highlighted areas are the standard errors. The gray dashed vertical line separates the contact and swing phase (average of seven participants with three gait cycle).

**Figure 3:**
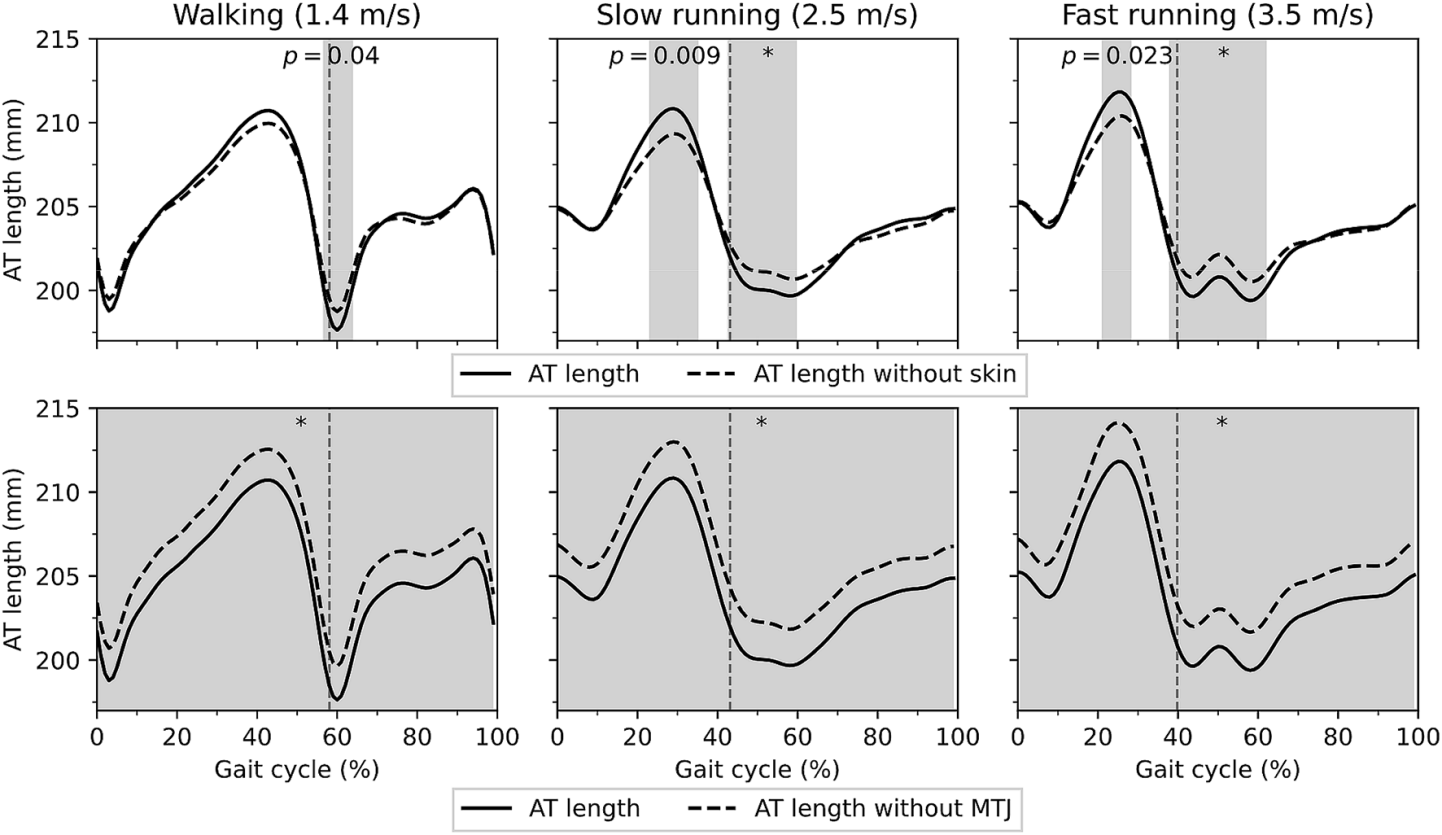
The first row shows the contribution of the skin-to bone displacement to AT length; the second row shows the contribution of the projection of the MTJ to the skin surface to AT length. AT length: The length of AT considering its curve-path shape, skin-to-bone displacement and the projection of the myotendinous junction (MTJ) to the skin surface. AT length without skin: AT length considering its curve-path shape and the projection of the MTJ to the skin surface. AT length without MTJ: The length of AT considering the curve-path shape and skin-to-bone displacement; the x-axis is normalized to the gait cycle. The filled gray areas indicated a significant difference between methods as result of the SPM paired t-test. The gray dashed vertical lines separate the contact and swing phase. The ‘*’ sign indicates the p-value < 0.001. Otherwise, the exact p-values are annotated in the figure. (the average of eleven participants with ten gait cycles). The maximum tendon force and elongation during the isometric MVCs were 5.523 ± 0.552 kN and 14.0 ± 2.5 mm, respectively. The adjusted r-square from the force–elongation of the quadratic equation (Eq. (5)) was, on average, 0.98 ± 0.01 (*p* < 0.001), and the average values of the two constants **a** and **b** were 8.7 and 181, respectively. Strain, force, strain energy, and the EMG activity of the three investigated muscles during walking and running are depicted in Fig. 4.

The non-normal distribution of all data was rejected significantly (*p* =  < 0.001). A significant gait speed effect was found on stance-time and cadence (*p* < 0.001) but not on swing-time (*p* = 0.436, Table 1). Comparing semi-automatic versus manual tracking of the MTJ position, the variance accounted for (VAF) were 93 ± 0.4%, 97 ± 0.1% and 97 ± 0.5%, and the adjusted r-squares were 0.96 ± 0.02, 0.99 ± 0.008 and 0.98 ± 0.02 (*p* < 0.001) during walking, slow running and fast running, respectively, evidencing high conformity of the developed algorithm with manual tracking (Fig. 2). The average root-mean-square error (RMSE) of the skin-to-bone displacement on the AT length during gait was between 0.92 and 1.09 mm. The average RMSE of the projection of the MTJ to the skin surface and AT length was between 1.9 and 2.1 mm (Table 2). The SPM-analysis (statistical parametric mapping) showed significant effects (*p* < 0.001) of both skin-to-bone displacement and the projection of the MTJ to the skin on the AT length-measurement during walking and running (Fig. 3). The significant differences between AT length and length without considering the skin-to-bone displacement were in walking, mainly at the beginning of the swing phase (Fig. 3). During running, there were significant differences in both stance and swing phases (Fig. 3). The effects of MTJ projection to skin were more prominent and expanded in the whole gait cycle (Fig. 3).

**Table 1:** Duration of the stance and swing phases and cadence during walking and running (average value ± standard deviation).

	Walking (1.4 m/s)	Slow running (2.5 m/s)	Fast running (3.5 m/s)
Stance (ms)*	605 ± 47^# ^^	330 ± 36^#^	279 ± 19
Swing (ms)	437 ± 33	436 ± 45	423 ± 32
Cadence (gait cycles/s)*	1.7 ± 0.1^# ^^	2.3 ± 0.1^#^	2.5 ± 0.1

*Statistically significant gait effect (p < 0.05).

^#^Statistically significant differences (post hoc analysis) to fast running (p < 0.05).

^^^Statistically significant differences (post hoc analysis) to slow running (p < 0.05).

**Table 2:** Contribution of skin-to-bone displacement and projection of MTJ to the skin surface on the AT length as means of the root mean square error (average value ± standard deviation).

	Walking (1.4 m/s)	Slow running (2.5 m/s)	Fast running (3.5 m/s)
Skin-to-bone (mm)	0.92 ± 0.57	1.08 ± 0.61	1.09 ± 0.53
MTJ projection (mm)	1.90 ± 0.90	2.00 ± 0.80	2.10 ± 0.80

**Figure 4:**
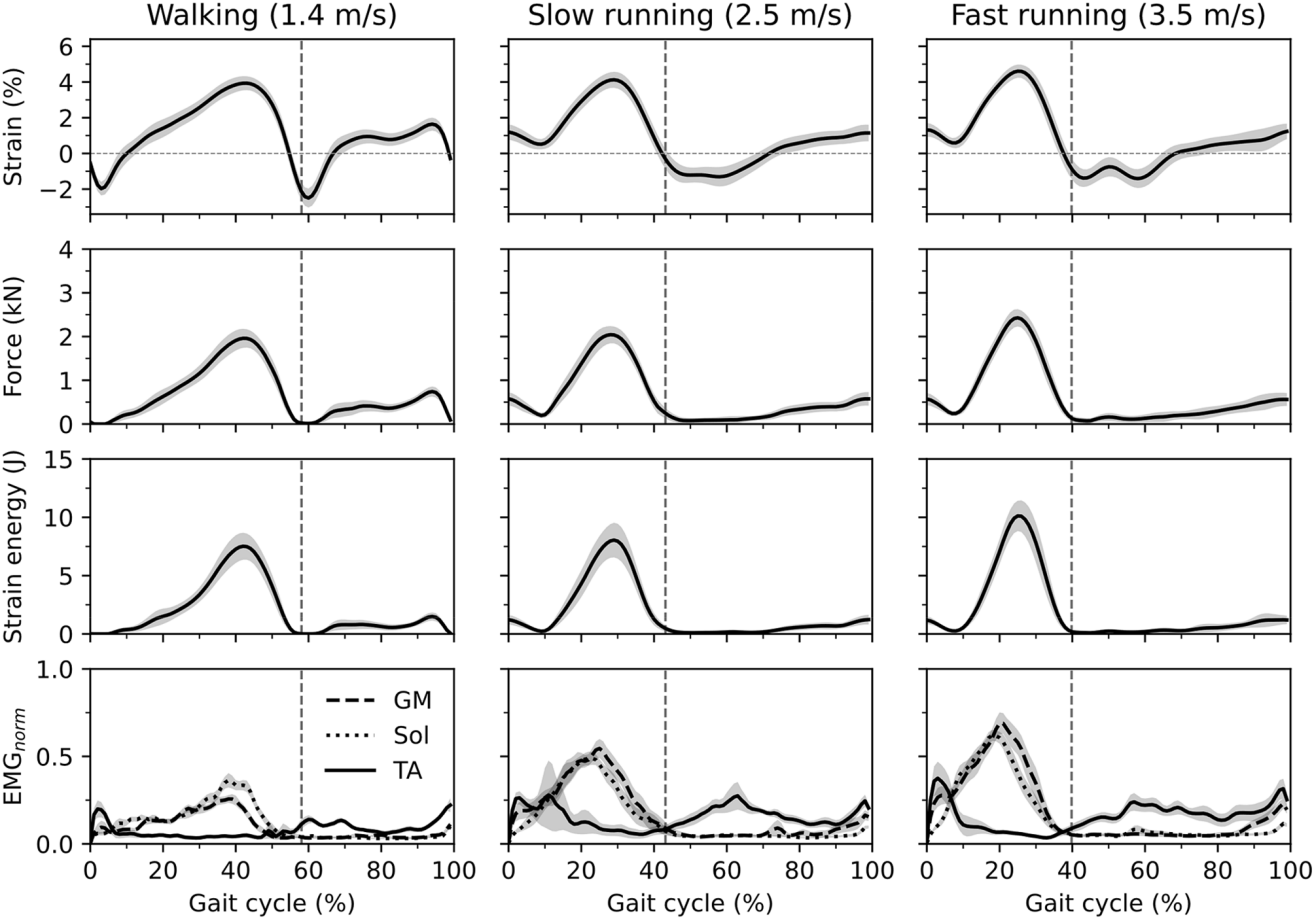
Achilles tendon (AT) strain, force and strain energy as well as EMG activity of the gastrocnemius medialis (GM), soleus (Sol) and tibialis anterior (TA) muscle during walking (1.4 m/s), slow running (2.5 m/s), and fast running (3.5 m/s). The gray highlighted area around the curves indicates the standard error. The x-axis is normalized to the gait cycle. The gray dashed vertical line separates the contact and swing phase (average of eleven participants with ten gait cycles).

We found a significant effect of gait speed on maximum AT strain (*p* = 0.043), force (*p* = 0.025), strain energy (*p* = 0.008) and strain energy recoil during the propulsion phase (*p* = 0.007, Table 3). The post hoc analysis showed significant differences in maximum strain, force, strain energy and energy recoil during the propulsion phase between walking and fast running (*p* = 0.023, *p* = 0.018, *p* = 0.016, *p* = 0.014, respectively) as well as between slow running and fast running (*p* = 0.023, p = 0.018, *p* = 0.016, *p* = 0.014, respectively). At the beginning of the stance phase, the initial strain energy recoil did not show significant differences (*p* = 0.635) between slow and fast running (Table 3).

**Table 3:** Peak values of strain, force and strain energy of the Achilles tendon (AT) as well as maximum normalized EMG activities of the gastrocnemius medialis (GM_norm_), soleus (Sol_norm_), and tibialis anterior (TA_norm_) during walking and running. Furthermore, elastic strain energy recoil during the propulsion phase and at the initial part of the stance phase is presented (average value ± standard deviation).

	Walking (1.4 m/s)	Slow running (2.5 m/s)	Fast running (3.5 m/s)
Strain_max_ (%)*	4.0 ± 1.2^#^	4.5 ± 1.3^#^	4.9 ± 1.2
Force_max_ (kN)*	1.989 ± 0.0684^#^	2.284 ± 0.0536^#^	2.556 ± 0.0540
Strain energy_max_ (J)*	7.8 ± 3.7^#^	9.5 ± 4.3^#^	11.3 ± 4.1
Propulsion recoil (J)*	7.8 ± 3.9^#^	9.1 ± 4.4^#^	11.3 ± 4.3
Initial recoil (J)	_	1.88 ± 1.10	1.71 ± 0.65
GM_norm_*	0.31 ± 0.09^# ^^	0.68 ± 0.15	0.8 ± 0.2
Sol_norm_*	0.38 ± 0.11^# ^^	0.61 ± 0.13^#^	0.73 ± 0.11
TA_norm_	0.27 ± 0.13	0.54 ± 0.55	0.5 ± 0.27

*Statistically significant gait effect (p < 0.05).

^#^Statistically significant differences (post hoc analysis) to fast running (p < 0.05).

^^^Statistically significant differences (post hoc analysis) to slow running (p < 0.05).

The maximum tibialis anterior (TA) EMG activity during the stance phase was not significantly different between the three gait speeds (*p* = 0.128). A significant effect of gait speed on maximum GM and soleus (Sol) EMG activity was found (*p* < 0.001). The pos thoc analysis revealed a significant difference between walking and slow running (*p* < 0.001) and walking and fast running (*p* < 0.001) for Sol and GM muscles. The post hoc comparisons between slow and fast running revealed a significant difference (*p* = 0.001) only for the Sol muscle (Table 3).

## Discussion

In order to assess the mechanical loading and strain energy of the AT during walking and running, we measured AT length using a new in vivo approach, which considers the tendon curve-path shape, taking into account the offset between MTJ and skin surface and the potential movements of the skin relative to the calcaneus bone at the AT insertion marker. Further, we developed a novel algorithm to track the MTJ position from the ultrasound image sequences that showed high conformity with a manual analysis. The of video frames analyzed for walking was on average 1912 ± 411 pictures and for running between 1324 and 1407 pictures for ten gait cycles. The average time needed for the manual tracking of one video was 7.9 h for walking and 5.4 h for running trials. The time for the analysis using our algorithm was considerably reduced to 17 min for walking and 13 min for running (i.e., including manual adjustment), giving evidence for essential improvements of the MTJ tracking's time-efficiency without a decline in the quality of the outcome.

The AT morphology features a variable curvature that changes during muscle contraction^33,34^. Therefore, the consideration of the AT curved shape is crucial for the investigation of in vivo conditions^35^. A reconstruction of the AT curvature using skin markers is a practical and valid approach to address this issue^27–29^. Our findings indicate that the misalignment of the MTJ and the markers on the skin as well as the skin-to-bone displacement are two important error sources that affect AT length measurements during locomotion when using this approach. The MTJ projection effect on the AT length was significant during the whole gait cycle in both walking and running. The RMS differences between AT length and AT length without MTJ projection, which depicts the contribution of the MTJ projection to the skin surface, were on average, 2.0 mm (across gait speeds), resulting in 1.01% of AT strain. The contribution of the skin-to-bone displacement was lower compared to the MTJ projection. However, the RMSE was 1 mm on average, indicating 0.45% inaccuracy on AT strain, thus a notable effect on AT length and strain.

As expected, the maximum strain values in all gait conditions occurred during the stance phase and close to the beginning of the propulsion phase. Shortly after take-off, strain drops rapidly to its minimum value and again increases with a moderate slope until the end of the gait cycle. The maximum strain values were, on average, between 4.0% during walking and 4.9% during running, corresponding to 51 and 62% of the strain achieved during the MVCs. Earlier studies^7,8^ reported maximum AT strain values of 3.9 to 4.4% during walking at velocities of 1.25 to 1.4 m/s. These values are very close to the maximum strain we found (4.0%) in the current study at 1.4 m/s walking velocities. During running, we depicted 4.5% to 4.9% of maximum AT strain at speeds of 2.5 and 3.5 m/s. Most of the earlier reports about the maximum AT strain during running are based on calculations using a simple planimetric model of the gastrocnemius or/and the soleus muscle–tendon unit^6,8,9^. In those planimetric models, the length of the tendon was calculated by subtracting muscle fascicle length projected in the direction of the line of force application from the MTU length. The reported maximum tendon strain values using the soleus muscle–tendon unit ranged from 6.0 to 8.0% at velocities from 2.0 to 4.0 m/s. For the gastrocnemius medialis muscle–tendon unit Lichtwark et al.^7^ found at 2.1 m/s running velocity maximum strain values of 5.5% where Monte et al.^6^ reported strains of 3.5 to 4.0% at running velocities of 2.8 and 3.6 m/s. Further, Lai et al.^9^ calculated maximum tendon strains of 2.0 to 3.0% at velocities of 2.0 to 4.0 m/s.

The repetitive strain of tendons is a crucial mechanical stimulus that regulates cell function. It affects the expression of growth factors and the synthesis of matrix proteins^16,36,37^, determining tendon plasticity^15,17,19^. Previous studies examining the effects of submaximal running on AT mechanical properties reported similar AT stiffness between runners and untrained individuals in young and old adults^20,21^ and no adaptive effects of long-time running training on the AT properties^24^. Considering that the effective mechanical stimulus for tendon adaptation in terms of strain magnitude ranges between 4.5 and 6.5% strain and long duration of loading (i.e., 3 s)^17,19,38^, our findings indicate that submaximal running does not provide sufficient tendon loading magnitude for triggering improvements of the AT mechanical properties and may explain the lack of observable effects in the former studies. The found maximum AT strain of 4.0 to 4.9% during the stance phase of walking and running was very short in duration. The time interval where AT strain exceeded 4.5% in five individuals during slow running and seven individuals during fast running on average 90 ± 40 ms for both running speeds. These results indicate a very short duration of AT strain above the reported threshold and less compared the recommended longer duration per repetition (i.e., 3 s) for an effective loading stimulus for tendon adaptation^39^. The average strain during the stance phase was for walking 1.9% and for running ranged from 2.2 to 2.4%, indicating low mechanical loading on the AT and insufficient to trigger additional adaptive responses. Therefore we can argue that in both conditions, walking and submaximal running, the applied mechanical loading on the AT is likely too low to initiate anabolic tendon responses^38^.

The assessed maximum AT force in the current study was for walking 2.7 and for running between 3.2 and 3.5-times of body weight. These values are significantly lower than predictions based on musculoskeletal models, which reported maximum AT forces of 3.9 of body weight during walking^12^ and between 5 and 7 of body weight during running^10,40^. These discrepancies might be a result of musculoskeletal model limitations, for example, the difficulties in the prediction of activation dynamics using optimization methods^41,42^ for muscle force predictions, which is more evident during rapid movements^43^. Further, the missing individual muscle and tendon properties for all involved muscles and the non-consideration of all possible forces that contribute to the resultant ankle joint moment (e.g., ligaments, bones) in the musculoskeletal models increase the model limitations.

It has been generally accepted that one of the primary roles of the tendon in vertebrates is to store energy during stretching and recoil during shortening^44,45^. Our results showed an average maximum elastic energy recoil during the propulsion phase of 7.8 J during walking, which increased to 9.5 and 11.3 J during slow and fast running. Although the pos thoc analysis resulted in a statistically significant difference of elastic strain energy recoil only in fast running compared to walking and slow running, the positive effect of gait speed on the energy storage and recoil during locomotion is notable and in agreement with earlier studies^5,6,8^. During the push-off phase of walking and running, almost the whole AT strain energy is returned to the human system (at take-off, the AT strain energy is very close to zero). It is well accepted that this recoil of energy reduces the plantar flexor muscles mechanical work^46^ to accelerate the body's center of mass in the desired movement direction^47^. The average assessed maximum AT force of 1.989 kN in the current study during walking is very close to the reported values measured with the optic fiber method (~ 1.7 kN) at a similar walking velocity^4^ and consequently, the elastic energy recoil is also close to our assessment. During running, we found AT tendon forces between 2.284 to 2.556 kN. These values are significantly lower compared to tendon force estimations using inverse dynamics and musculoskeletal model approaches (3.06 to 4.64 kN^10,11^). These differences result in differently calculated tendon strain energy storage and recoil values. In our study and using the experimentally assessed quadratic function of the tendon force–length curve, we found significantly lower values in the energy recoil during the propulsion phase of running than studies using an inverse dynamics approach and musculoskeletal modeling^5^. Lai et al.^5^ reported an AT energy recoil during the propulsion phase of 27.7 and 38.7 J, at running speeds of 2.1 and 3.5 m/s, respectively. These values are about three times higher than in the current study with 9.2 and 11.2 J at 2.5 and 3.5 m/s of running speed, respectively. In the inverse dynamics approach by Lai et al.^8^, the passive forces transmitted by ligaments, by bone-to-bone contacts and by soft tissues around the ankle joint are not considered, and therefore the calculated muscle forces might be overestimated, resulting in an overestimation of the tendon strain energy.

Our results also demonstrated elastic strain energy recoil directly after the touchdown during running. The recoil of strain energy in this initial stance phase (i.e., 70 to 76 ms) was between 1.7 and 1.9 J. These values are 15 to 20% of the maximum AT strain energy during the stance phase. Therefore, they might be functionally relevant. We can exclude that this initial strain energy recoil might be dissipated by the muscle contractile elements of the triceps surae muscle because in the investigated running velocities, the fascicles of all three muscles of the triceps surae show an active shortening^5,7,32^. We interpret this finding as the recoil of AT tendon elastic strain energy to the body in this initial phase of stance right after touchown. This phenomenon (i.e., a decrease of AT force and recoil of elastic strain energy after heel contact) has not been mentioned in the literature, and, therefore, the functional consequences for the running task are not known. Komi et al.^31^, using a force transducer that was surgically implanted in the AT, reported a decrease of AT force directly after the touchdown in rearfoot running and suggested an association with the reduction of TA EMG activity. As the foot contacts the ground, the TA muscle showed a high EMG activity, indicating an active control of the initial plantar flexion through muscular force. The reduction of the AT force after heel contact decreases the internal resultant ankle joint moment, supporting the TA function as a regulator of the ankle joint during the initial part of the stance phase. However, it is difficult to explain the resulting functional consequences of the AT elastic energy recoil in this initial phase because it is unclear where this energy is returned. A recoil of AT strain energy must not necessarily increase the mechanical work/power at the ankle joint but can, for example, be absorbed by the TA tendinous tissues^48^ or by the elastic structures of the foot arch^49,50^ and stored as elastic strain energy.

We measured the force–elongation relationship of the AT using MVCs with a slow rate of the force application. The participants completed five trials of isometric ramp contractions, steadily increasing effort from rest to the maximum in ~ 5 s resulting in an average tendon loading rate of 1.076 ± 0.456 kN/s. Using the individually assessed force–elongation relationship, we calculated the AT force during walking and running. During the stance phase in the investigated conditions, the ATloading rate ranged from 16.5 to 20.0 kN/s and was significantly higher compared to the MVCs. Tendons, as biomaterials, are viscoelastic, and, therefore, the differences in the loading rate may influence the accuracy of the tendon force assessment during the walking and running trials. However, tendon hysteresis is ~ 10%^51,52^, indicating minor damping components in the tendon properties. Ker et al.^53^ found that loading frequencies from 0.22 to 11 Hz (loading rate from 0.580 to 31.240 kN/s) did not affect the youngs modulus of the tendon and, therefore, we can assume a negligible effect of loading rate on tendon dynamics during physiological activities like walking and running. More recently, Rosario and Roberts et al.^54^ investigated tendon strains at loading rates between 10 and 80 MPa/s and predicted a change from slow to fast loading of 0.16% strain on the human AT during running, evidencing a minor effect of loading rate on tendon dynamics. Based on these reports, we can argue that the loading rate differences between the MVCs and the investigated locomotor activities did not significantly affect the AT force and strain energy assessment.

**Figure 5:**
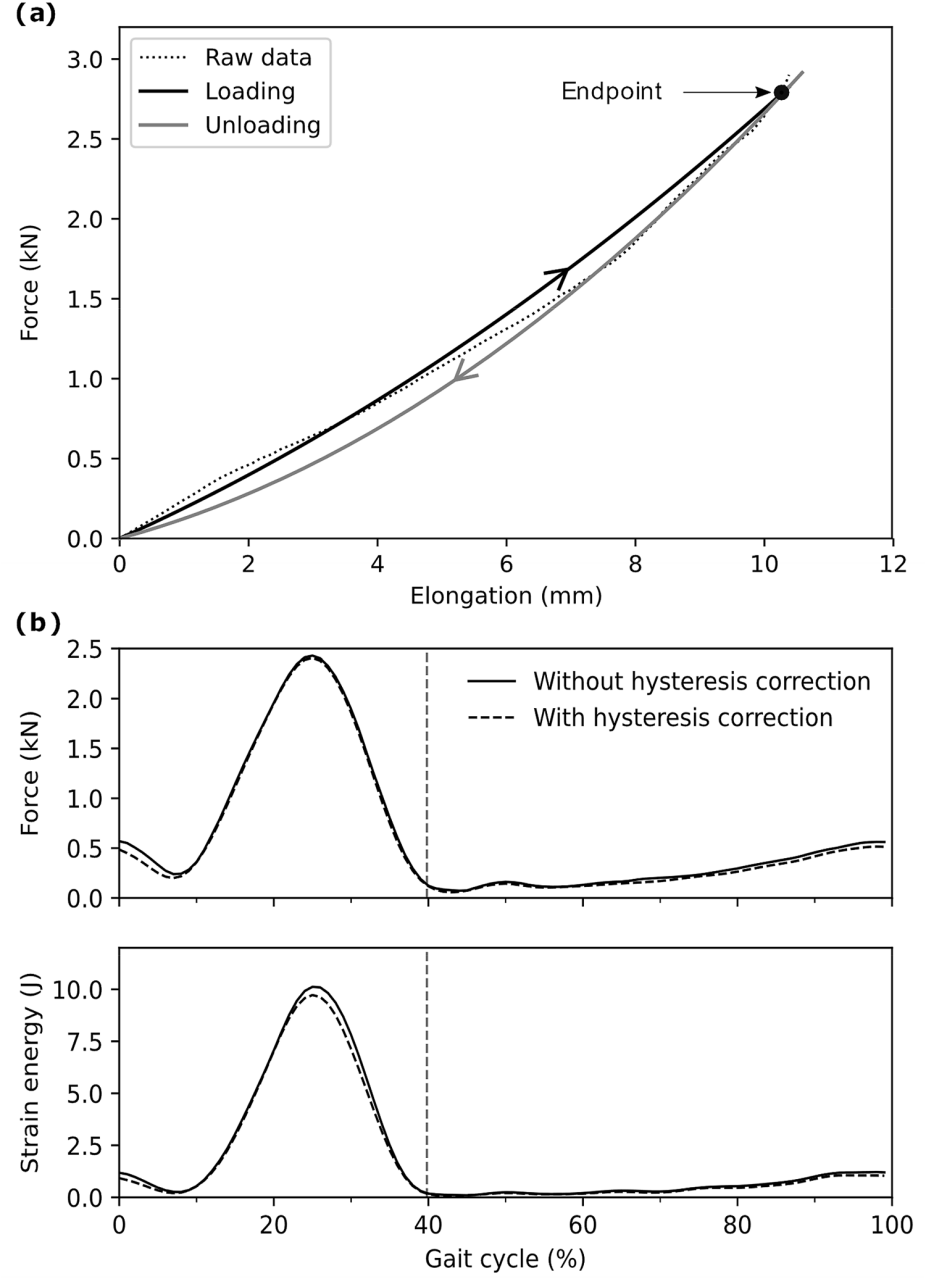
(**a**) Experimentally assessed Achilles tendon (AT) force–elongation relationship during maximum voluntary contractions (Raw data) and the modeled force–elongation curve during loading and unloading (eleven individuals with ten gait cycles. (**b**) AT force and AT strain energy with and without hysteresis consideration during fast (3.5 m/s) running. The x-axis is normalized to the gait cycle. The gray dashed vertical line separates the contact and swing phase.

Further, both the loading and unloading phases of AT force were assessed from the same force–elongation curve during MVC trials, and the effect of hysteresis in the unloading phase was not considered. To examine the impact of the hysteresis effect on both AT force and AT strain energy, we assumed a 10% hysteresis, as it is the reported order of magnitude in most studies^51–53^. For that reason, we determined the two coefficients (a and b) of the Eq. (5) for the unloading phase based on the following constraints: (a) force–elongation curve during the unloading phase will reduce the strain energy by 10%, and (b) both force–elongation curves (loading and unloading) will end in the same point (Fig. 5a). The two constant coefficients of the unloading curve were reached by solving two equations with two unknown variables. The choice of appropriate coefficients in the recalculation of force and strain energy was based on the first derivative of the AT length. If the first derivative was negative, the unloading curve's coefficients were used; otherwise, the standard force–elongation curve was used (Fig. 5b). The contribution of hysteresis to AT force and strain energy was assessed using RMSE, which resulted in a negligible effect of 46 N and 0.2 J in forces and energy, respectively (i.e., 1.9% of the maximum AT force or strain energy).

In conclusion, in this study, we introduced a new in vivo assessment of the AT mechanical loading and strain energy during locomotion, demonstrating that when taking into account the curvature of the AT using skin markers, the projection of the MTJ to the skin and skin-to-bone displacement are two methodological issues that significantly influence the AT length measurement. We found that the AT mechanical loading during submaximal running is lower than previously reported and inadequate to initiate an adaptation of tendon mechanical properties, which explains at least partly the reported absence of significant differences in the AT mechanical properties between runners and non-runners. Finally, we provided the first evidence of an elastic strain energy recoil at the beginning of the stance phase during running, which might be functionally relevant for running economy.

## Methods

### Experimental design

Eleven adults (one female) with an average height of 177 $\pm$ 6 cm, body mass of 74 $\pm$ 9 kg, and age 29 $\pm$ 3 years participated in this study. All participants gave written informed consent to the experimental procedure, which was approved by the ethics committee of the Humboldt-Universität zu Berlin (HU-KSBF-EK_2018_0005) and followed the standards of the Declaration of Helsinki. The participants were in full health, and none of them reported neuromuscular or skeletal impairments in the past year. After a familiarization phase, the participants walked (1.4 m/s) and ran (2.5 m/s and 3.5 m/s) with randomized order on a treadmill (Daum electronic, ergo_run premium8, Fürth, Germany) whereby AT length was determined experimentally by integrating kinematics and ultrasound analysis. AT length was defined as the distance between the origin (i.e., the most distal junction) of the GM and the insertion on the tuber calcanei.

During gaits, marker-based motion capture was used to track the position of the insertion point, represented by a marker that was placed over the tuber calcanei as well as joint kinematics (Fig. 1a). The potential skin-to-bone displacement was assessed while the foot was passively rotated (5°/s) throughout the full range of motion from plantar flexion to dorsiflexion by means of ultrasonography (Fig. 1b). Ultrasound was used to detect the MTJ, and the transducer position was tracked by the motion capture system using a mounted marker tripod (Fig. 1a). To assess the AT's curved path, small foil markers were placed on the skin covering the AT path on the line from the defined origin to the insertion (Fig. 1a). The MTJ position was then projected to the skin surface (Fig. 1a) and mapped to the global coordinate system to assess AT length during gait (i.e., from projected MTJ over the curved foil marker path to the insertion). Force and strain energy of AT during locomotion were assessed by fitting a quadratic function to the experimentally measured force–length curve of the AT based on individual maximum voluntary isometric contractions (MVC). During gait, the AT force was assessed by applying coefficients of the quadratic function to the AT elongation values obtained during locomotion. The AT tendon strain energy during walking and running was calculated by integrating tendon force over tendon elongation.

### Kinematics and gait cycle determination

Kinematic data of the right leg were assessed using six reflective markers (14 mm in diameter) placed on the tip of the toe, medial and lateral epicondyle, on a line from greater-trochanter to lateral epicondyle as well as medial and lateral malleolus. 3D-trajectories of all markers were captured in real-time with 14 Vicon (Version 1.7.1, Vicon Motion Systems, Oxford, UK) cameras (4× MX T20, 2× MX-T20-S, 6× MX F20, 2× MX F40, 250 Hz). A fourth-order low pass and zero-phase shift Butterworth filter with a cut-off frequency of 12 Hz was applied to the raw marker trajectories (including foil markers). A one-minute warm-up and familiarization phase with the treadmill in each of the three speeds was considered before the captured trials, which included at least twelve stride cycles for each speed. During walking, the foot touchdown was determined as the heel marker's instant minimal vertical position and during running as the first peak of the knee joint angle (i.e., extension)^55^. The take-off was defined as the reversal of the toe marker's anterior–posterior velocity during walking and as the second peak of the knee joint angle during running.

### Measurement of the AT length during gait

The point of insertion of the AT, defined at the notch of the tuber calcanei of the calcaneus bone^56^, was detected in sagittal plane ultrasound scans. The origin of the AT was determined as the most distal position of the GM MTJ, obtained by transversal and sagittal ultrasound scans. A T-shaped 60 mm ultrasound transducer (Aloka UST-5713T, Hitachi Prosound, alpha 7, Japan) operating at 146 Hz was fixed over the GM MTJ with a customized, flexible plastic cast. A gel pad was used to account for surface unevenness. The ultrasound device was time-synchronized with the motion capture system using a manual analog trigger signal. The AT curved path was elaborated by placing reflective foil markers on the skin that directly cover the AT^28,29^. Depending on the position of the MTJ on the shank length, varying number (i.e., from 5 to 9) of reflective plane foil markers with 5 mm in diameter and 20 mm interval gap were placed on the path of AT from the defined insertion point to the last possible position below the plastic cast. Note that the ultrasound probe was then oriented in extension to this line. The curved path's length was calculated as the sum of the vectors, which was defined by the position of two consecutive foil markers.

An image-based tracking algorithm was developed to determine the position of the MTJ from the ultrasound videos (MATLAB, version 9.6. Natick, Massachusetts: The MathWorks Inc). The procedure included a multi-updating template-matching technique with 33 manually defined templates. These 33 templates were rectangular windows covering the area around the MTJ and were defined in the first gait cycle, distributed equally in time throughout the stance and swing phase. The created templates then served to detect the MTJ during the subsequent steps (10 step cycles of the right leg on average). The ultrasound images were first cropped to a region of interest (in the range of MTJ displacement) and were convolved with a gaussian (3 $*$ 3 kernel size) filter to reduce noises. By sweeping each template on the cropped image, the maximum value of normalized 2D cross-correlation was detected as the position of MTJ. Each template auto-updates itself under specific criteria until the next manually defined template occurs. The criteria of the auto-updating template were defined as Eqs. (1) and (2):

1 $${\mathrm{d}}= \sqrt{{\left({X}_{n}-{X}_{n-1}\right)}^{2}-{\left({Y}_{n}-{Y}_{n-1}\right)}^{2}}<{T}_{1}$$

2 $$D= \sqrt{{\left({X}_{n}-{X}_{c}\right)}^{2}-{\left({Y}_{n}-{Y}_{c}\right)}^{2}}<{T}_{2}$$

where ${{\varvec{X}}}_{{\varvec{n}}}$ and ${{\varvec{Y}}}_{{\varvec{n}}}$ are the coordinates of the best-matched position (MTJ coordination) in the current frame, ${{\varvec{X}}}_{{\varvec{n}}-1}$ and ${{\varvec{Y}}}_{{\varvec{n}}-1}$ are the best-matched position in the previous frame, ${{\varvec{X}}}_{{\varvec{c}}}$ and ${{\varvec{Y}}}_{{\varvec{c}}}$ are the coordination of best-matched position in the last accepted frame (i.e., the last frame that template was auto-updated), ${{\varvec{T}}}_{1}$ and ${{\varvec{T}}}_{2}$ are the thresholds defined in pixel (in our case was 5 and 10 pixels, respectively), ***d*** is the Euclidean distance between the best-matched position of the current frame and the previous, **D** is the Euclidean distance of the matched position between the current frame and the last accepted frame. If ${\varvec{d}}$ and ${\varvec{D}}$ were below the thresholds, then the updated template was accepted and used for the next frame. If the criteria were not fulfilled, the last accepted template was used for the next frame. Visual inspection of all frames was conducted afterward to verify the automatic tracking results, and corrections were made if necessary. When an inappropriately tracked MTJ position was deleted, the gap was filled by linear interpolation. In case the interpolation was unacceptable, the position of the MTJ was defined manually.

A custom 3D-printed marker tripod was fixed to the ultrasound transducer. The calibration of the ultrasound images with respect to the tripod was done by digitizing the four corners of the transducer's protective front layer. A coordinate system was defined (P) on the center-left side of the protective front layer. The gap between the left edge side of the protective front layer to the first piezoelectric sensor underneath was determined by subtracting the plate's width from the ultrasound image's width divided by two. This gap size was then verified with an x-ray image of the transducer. The coordinate system (t) on the transducer (defined by a mounted tripod) was then adjusted accordingly. Another coordinate system (2D) was determined with the origin located at the first pixel (top left) of the ultrasound image (U). To map the MTJ position from the ultrasound image to the global coordinate system, a global transformation matrix (${}_{U}^{V}T$) was used. This matrix was created by multiplicating the three transformation matrixes in Eq. (3)^57^.

3 $${}_{U}^{G}T= {}_{t}^{G}T \times {}_{P}^{t}T \times {}_{U}^{P}T$$

${}_{U}^{P}T$ is the transformation matrix to transform the coordinate systems from the ultrasound image [U] to the protective front face of the transducer [P], ${}_{P}^{t}T$ is to transform the coordinate system of the protective front face of the transducer to tripod [t] and ${}_{t}^{V}T$ transfer the tripod coordinates to the global system [G] (i.e., defined by the motion capture system). In this way, the detected MTJ position from the ultrasound images could be projected to the global coordinate system by Eq. (4):

4 $${P}_{V}= {}_{U}^{G}T \times \left[\begin{array}{c}\begin{array}{c}{P}_{ux} \cdot scale\\ {P}_{uy} \cdot scale\\ 0\\ 1\end{array}\end{array}\right]$$

where ${P}_{V}$ is the transformed MTJ position to the global coordinate system, ${P}_{ux}$ and ${P}_{uy}$ are the detected MTJ position in the $\mathrm{vertical}$ and horizontal direction in the ultrasound images, respectively, and *scale* is the pixel to millimeter scale-factor for the US image (i.e., 4.3 in our case). For the projection of the MTJ to the skin surface, a double threshold-to-intensity gradient of the image was utilized. Then a spline curve was fitted to the detected edges of the skin surface, and the position of the MTJ was transferred to the fitted curve via the shortest distance.

The kinematic and ultrasound recordings obtained to account for potential displacements of the reflective calcaneus marker above the defined insertion point (notch at tuber calcanei) were captured in two separate sessions due to spatial constraints around the ankle (i.e., only a reflective marker or an ultrasound probe could be mounted above the calcanei at the same time). In the first session, the participants lay prone on a dynamometer bench (Biodex Medical, Syst. 3, Inc., Shirley, NY) while their foot was fixed to the dynamometer footplate. Three trials were recorded while the foot was rotated passively by the dynamometer (5°/s) from 30° plantar flexion to the individual maximum dorsiflexion angle (Fig. 1b). A sound-absorptive skin-adhesive marker was placed about 5 mm above the identified insertion point on the skin. A three-centimeter ultrasound transducer (My Lab60, Esaote, Genova, Italy, 37 Hz) was attached above the calcaneus bone over the insertion of the AT in a sagittal plane. The skin position was detected in the ultrasound images by registering a shadow line as the sound absorptive marker's effect, and the notch identified the bone's position as a fixed bony landmark (Fig. 1b). The skin position relative to the bone was tracked throughout the full range of motion from plantar flexion to dorsiflexion. In the second session, the ultrasound probe was removed, and reflective markers were placed precisely on the same positions as during the treadmill session, i.e., epicondyle (lateral and medial), malleoli (lateral and medial), between the first and the second metatarsal and on the defined insertion point (i.e., notch). Then the ankle joint was again passively rotated under the same conditions by the dynamometer. The heel angle (i.e., the created angle between the two vectors, first the knee joint center and ankle joint center and the second the heel and ankle joint center) was calculated and matched to the angle given by the dynamometer observed in the first session. Finally, the differences between skin and bone positions measured in the ultrasound images were converted to millimeters, and a spline curve was fitted to the skin-to-bone displacement versus heel angle (Fig. 1c). The individual error model was then used to correct potential skin-to-bone movements as a function of the heel angle during gaits. The average maximum displacement of the skin relative to the bone was 2.30 ± 0.95 mm in plantar flexion and 1.90 ± 0.69 mm in dorsiflexion during the ankle's passive rotation.

### Measurement of muscle electromyographic activity

Surface electromyographic (EMG) data of the TA, GM and Sol was measured during walking and running using a wireless EMG system (Myon m 320RX, Myon AG, Baar, Switzerland) operating at a sampling frequency of 1000 Hz. The EMG signal was processed using a fourth-order high-pass Butterworth zero-phase shift filter with a 50 Hz cut-off frequency, a full-wave rectification, and a low-pass zero-phase shift filter of 20 Hz cut-off frequency. The resultant EMG signal was then normalized to the maximum processed EMG obtained during individual MVCs.

### Assessment of AT strain, force and energy during gait

AT strain during walking and running was calculated by dividing the measured AT length by the AT resting length determined during the relaxed state in 20° plantar flexion, where AT slackness has been reported previously^28^. A force–elongation relationship of the AT was determined in a separate experiment, combining dynamometry and ultrasound measurements to calculate AT's force and strain energy during locomotion. Participants performed five isometric plantar flexion ramp MVCs (~ 5 s gradual increase of force) while their knee was fully extended, and the ankle angle at rest was set to the neutral position (tibia perpendicular to sole). Misalignments of the ankle axis of rotation and dynamometer axis during the MVCs, as well as gravitational and passive moments, were considered through inverse dynamics^58^. The effect of antagonistic muscle co-activation on the resultant joint moment during the MVCs was taken into account with an established procedure^59^. During contractions, the AT force was calculated by dividing the ankle joint moment by the AT's lever arm, which was determined using the tendon-excursion method^60^. As suggested previously, the tendon lever arm changes during the contractions were corrected^61^. The corresponding elongation of the AT during the five trials for each participant was assessed with a 10 cm linear ultrasound probe fastened over the GM MTJ. The position of the MTJ visualized by ultrasound was tracked in the ultrasonographic images with the semi-automatic tracking algorithm described above. The effects of unavoidable ankle joint rotation during the MVCs that cause displacements of the MTJ on the tendon elongation was corrected by subtracting the MTJ displacement tracked during a passive rotation of the ankle (full range of motion at 5°/s)^29^ with respect to the ankle joint angle changes during the MVCs. The tendon force–elongation relationship of the five trials of each participant was averaged to achieve excellent reliability. A quadratic function (Eq. (5)) was fitted to obtain the individual force–elongation relationship of the AT and then was used to assess AT force during gaits:

5 $$F = a* {l}^{2}+ b*l$$

where **F** is the AT's force during gaits, **a** and **b** are quadratic function coefficients, and ***l*** is the elongation of the AT during gait. The AT force's strain energy during walking and running was calculated by integrating the AT force over the measured AT elongation (we omitted the elongations below the resting length) using Eq. (6).

6 $$E=\int F. dl = \int \left(a*{l}^{2}+ b*l\right). dl = \frac{1}{3}a.{l}^{3}+ \frac{1}{2}b.{l}^{2}+c$$

where **E** is the AT's strain energy during gait, and **c** is the constant of integration. The maximum value of the AT strain energy during the stance phase is the total amount of elastic energy stored. Tendon energy recoil during the propulsion phase of walking and running was calculated as the difference between the AT maximum strain energy and the energy at take-off. The AT's elastic energy recoil at the beginning of the stance phase of running was calculated as the difference of the AT strain energy between the touchdown and its first local minimum after touchdown.

### Statistics

The semi-automatic MTJ tracking algorithm's validity was tested by comparison to manual tracking (i.e., an experienced research assistant student did all the manual tracking of the MTJ). Displacements of the MTJ position in the longitudinal direction were assessed with the parameter VAF and the Pearson correlation coefficient (r) for seven participants in three gait cycles of all gait speeds. SPM one-way repeated measures analysis of variance (ANOVA) was used to find if there is a main effect of skin-to-bone displacement or projection of MTJ to the skin surface to AT length. If a significant main effect was found, an SPM paired t-test was used to investigate the significant difference between methods. In addition to SPM, we also used RMSE between different methods of AT length calculations.

A one-way repeated-measures analysis of variance (ANOVA) was performed to examine the effects of gait speed on maximum AT strain, force and strain energy as well as strain energy recoil during the propulsion phase and during the initial part of the stance phase. The same ANOVA has been used for the EMG activity in GM, Sol and TA and temporal gait parameters such as stance-time, swing-time and cadence. A pairwise t-test with Benjamini–Hochberg corrected p-values were used for pos thoc analysis (adjusted p-values are reported) in case of significant main effects. The normal distribution of the data was tested with the Shapiro–Wilk test. The level of significance for all statistical tests was set to ${\upalpha }$ = 0.05.

## Data Availability

The datasets generated and analyzed during the current study are available from the corresponding author on reasonable request.
